# Namodenoson in Advanced Hepatocellular Carcinoma and Child–Pugh B Cirrhosis: Randomized Placebo-Controlled Clinical Trial

**DOI:** 10.3390/cancers13020187

**Published:** 2021-01-07

**Authors:** Salomon M. Stemmer, Nebojsa S. Manojlovic, Mihai Vasile Marinca, Petar Petrov, Nelly Cherciu, Doina Ganea, Tudor Eliade Ciuleanu, Ioana Adriana Pusca, Muhammad Shaalan Beg, William T. Purcell, Adina-Emilia Croitoru, Rumyana Nedyalkova Ilieva, Sladjana Natošević, Amedeia Lavinir Nita, Dimitar Nikolaev Kalev, Zivit Harpaz, Motti Farbstein, Michael H. Silverman, David Bristol, Inbal Itzhak, Pnina Fishman

**Affiliations:** 1 Davidoff Cancer Center, Rabin Medical Center-Beilinson Hospital, Petah Tikva and Sackler Faculty of Medicine, Tel Aviv 49100, Israel; shtemers@clalit.org.il; 2 Department of Gastroenterology and Hepatology, Military Medical Academy, 11000 Belgrade, Serbia; nebojsa.manojlovic1@gmail.com; 3 Department of Oncology, Iasi Regional Oncology Institute, Institutul Regional de Oncologie Iasi—Sectia Oncologie Medical, 700483 Iasi, Romania; marincarct@gmail.com; 4 Department of Medical Oncology and Oncological Diseases in Pneumology, Complex Oncology Center–Plovdiv, EOOD, 4000 Plovdiv, Bulgaria; petar_petrov_doctor@abv.bg; 5 Oncology Department, Clinica Onco-Life, 200255 Craiova, Romania; cherciu.nelly@yahoo.com; 6 Medical Oncology Department, Sf. Ioan Cel Nou County Clinical Emergency Hospital, 720224 Suceava, Romania; motan_doina@yahoo.com; 7 Institute of Oncology, University of Medicine and Pharmacy, 400015 Cluj-Napoca, Romania; vasilika_mariana@yahoo.com; 8 Oncology Department, S.C. Pelican Impex S.R., 410469 Oradea, Romania; ioana_covaciu@yahoo.com; 9 Division of Hematology and Medical Oncology, the University of Texas Southwestern Medical Center, Dallas, TX 75390, USA; muhammad.beg@utsouthwestern.edu; 10 Division of Medical Oncology, University of Colorado School of Medicine, Aurora, CO 80045, USA; tom.purcell@ucdenver.edu; 11 Oncology Department, Fundeni Clinical Hospital, 022328 Bucharest, Romania; adina.croitoru09@yahoo.com; 12 Department of Medical Oncology, Multiprofile Hospital for Active Treatment Central Onco Hospital OOD, 4000 Plovdiv, Bulgaria; md_rumi@abv.bg; 13 Oncology Department, Zdravstveni Centar Kladovo, 19320 Kladovo, Serbia; snatosevic@gmail.com; 14 Oncology Department, County Hospital Prahova, 100001 Ploiesti, Romania; amedeianita@yahoo.com; 15 Oncology Department, Sveta Marina University Hospital, 9002 Varna, Bulgaria; dimitar@kalevi.eu; 16 R&D, Can-Fite BioPharma, 10 Bareket St., P.O.Box 7537, Petah-Tikva 49170, Israel; zivit@canfite.co.il (Z.H.); Motti@canfite.co.il (M.F.); msilverman@biostrategics.com (M.H.S.); drbris1@gmail.com (D.B.); inbal@canfite.co.il (I.I.)

**Keywords:** Child–Pugh B, hepatocellular carcinoma, namodenoson, overall survival, randomized clinical trial

## Abstract

**Simple Summary:**

We conducted a phase II randomized placebo-controlled trial to investigate namodenoson, an A3 adenosine-receptor agonist, as 2nd-line treatment for advanced hepatocellular carcinoma and moderate hepatic dysfunction (Child–Pugh [CP] scores of 7–9). The study included 50 patients in the namodenoson arm and 28 patients in the placebo arm. No overall survival advantage was observed with namodenoson; however, in patients with CP score of 7 (34 namodenoson-treated, 22 placebo-treated), namodenoson was associated with a significant improvement in 12-month overall survival (44% versus 18%, *p* = 0.028). Response rates were determined in patients with ≥1 assessment post-baseline (34 namodenoson-treated, 21 placebo-treated). Partial response was achieved by 3 (8.8%) namodenoson-treated and 0 (0%) placebo-treated patients. Namodenoson was well-tolerated, with a safety profile comparable to that of the placebo group. No treatment-related deaths were reported. In conclusion, namodenoson demonstrated a favorable safety profile and a preliminary efficacy signal in the evaluated population.

**Abstract:**

Namodenoson, an A3 adenosine-receptor agonist, showed promising results in advanced hepatocellular carcinoma (HCC) and moderate hepatic dysfunction (Child–Pugh B; CPB) in a phase I/II clinical study. This phase II study investigated namodenoson as second-line therapy in such patients. Patients were randomized 2:1 to twice a day (BID) namodenoson (25 mg; *n* = 50) or placebo (*n* = 28). The primary endpoint (overall survival [OS]) was not met. Median OS was 4.1/4.3 months for namodenoson/placebo (hazard ratio [HR], 0.82; 95% confidence interval [CI] 0.49–1.38; *p* = 0.46). Pre-planned subgroup analysis of CPB7 patients (34 namodenoson-treated, 22 placebo-treated) showed a nonsignificant improvement in OS/progression-free survival (PFS). OS: 6.9 versus 4.3 months; HR, 0.81; 95% CI: 0.45–1.43, *p* = 0.46. PFS: 3.5 versus 1.9 months; HR, 0.89; 95% CI: 0.51–1.55, *p* = 0.67 (log-rank test). The difference in 12-month OS was significant (44% versus 18%, *p* = 0.028). Response rates were determined in patients for whom ≥ 1 assessment post-baseline was available (34 namodenoson-treated, 21 placebo-treated). Partial response was achieved by 3/34 (8.8%) and 0/21 (0%) patients, respectively. Namodenoson was well-tolerated, with a safety profile comparable to that of the placebo group. No treatment-related deaths were reported; no patients withdrew due to toxicity. In conclusion, namodenoson demonstrated a favorable safety profile and a preliminary efficacy signal in HCC CPB.

## 1. Introduction

Liver cancer is the fifth most common cancer worldwide and the second most frequent cause of cancer-related death globally, with an estimated 854,000 new cases and 810,000 deaths annually [1]. Hepatocellular carcinoma (HCC) accounts for approximately 90% of primary liver cancer cases and constitutes a major global health problem [1].

Patients with advanced HCC and Child–Pugh B (CPB) cirrhosis have a borderline liver function, and therefore the benefit of any therapy might be offset by the decline in their liver function. The only curative option for these patients involves successful downstaging and liver transplantation. However, this approach is appropriate only for a minority of patients and is further limited by the restricted number of livers available for transplantation [2]. The most common treatment for HCC and CPB is the multikinase inhibitor sorafenib, which is approved by the US Food and Drug Administration (FDA) for advanced HCC regardless of liver function [3].

Interestingly, all studies investigating therapeutic agents as first-line agents in advanced HCC enrolled only patients with Child–Pugh A (CPA). Similarly, in the second-line setting (after prior treatment with sorafenib), therapies are also primarily limited to CPA patients. CPB patients are generally excluded from clinical studies due to their poor prognosis and low expected response rate [4]. As of 12 July 2020, clinicaltrials.gov included 110 enrolling/active phase II or III clinical studies in advanced HCC [5]. Of these studies, the eligibility criteria of only 2 studies, in addition to the study reported herein, do not exclude CPB patients. Thus, clearly, therapies for HCC and CPB cirrhosis are still needed.

The Gi protein-coupled A3 adenosine receptor (A3AR) is overexpressed in different types of solid tumors, including melanoma, breast, colon, and prostate cancer, and HCC, whereas low receptor expression is found in adjacent normal tissues [6,7]. The high receptor expression is reflected in A3AR overexpression in the peripheral blood mononuclear cells (PBMCs) of patients with HCC, suggesting that receptor status in the remote tumor organ is mirrored in the PBMCs [6].

Namodenoson, generically known as Cl-IB-MECA, is a highly selective orally bioavailable A3AR agonist that induces an apoptotic effect towards HCC in syngeneic orthotopic and xenograft experimental animal models. Its mechanism of action entails deregulation of the NF-κB and the Wnt signaling pathways, followed by an increase in pro-apoptotic proteins and Fas-ligand, resulting in tumor growth inhibition [6,8]. A3AR, similar to other G protein receptors, is internalized upon agonist binding, degraded in the cytoplasm and subsequently re-synthesized and re-cycled into the cell membrane [9]. Preclinical pharmacologic studies showed that after chronic treatment of tumor-bearing animals with namodenoson, A3AR was downregulated shortly after treatment; however, A3AR levels resumed pretreatment levels after a few hours [9]. Moreover, namodenoson induces differential effects on tumor and normal cells, acting as a protective agent in the liver by preventing normal hepatocyte apoptosis as well as protecting against hepatic ischemia/reperfusion injury following partial hepatectomy [8,10].

In a previously encouraging open-label phase I/II trial, the safety and efficacy of namodenoson were assessed in patients with advanced unresectable HCC, 67% of whom failed prior sorafenib treatment [11]. Median overall survival (OS) was 7.8 months for the whole study population, and for CPB patients (28%), it was 8.1 months. Namodenoson was safe and well-tolerated, and a direct correlation between A3AR expression levels at baseline and tumor response to namodenoson was found [11].

The current randomized, placebo-controlled, phase II study evaluated the efficacy/safety of namodenoson versus placebo in advanced HCC CPB patients. In addition, we evaluated the presence of the A3AR target during the treatment period.

## 2. Results

**Figure 1:**
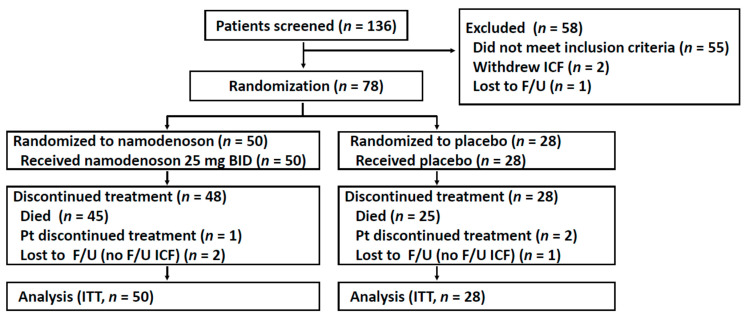
Flow chart of patient recruitment, treatment, and follow-up. F/U, follow-up; ICF, informed consent form; ITT, intention to treat; Pt, patient.

Between December 2014 and August 2017, 136 patients were screened, of whom 78 patients were enrolled and randomized (50 were to namodenoson, 28 to placebo, Figure 1). Due to unexpectedly prolonged survival in several patients, the decision was made to unblind the trial (in March 2019) and perform the survival analysis after 70 deaths had occurred. The baseline patient/tumor characteristics of the study population are presented in Table 1. Age, sex, and ethnicity were generally balanced between the treatment groups and consistent with characteristics observed in similar trials. All patients had previously received sorafenib. The majority of patients (71.8%) presented with a Child–Pugh (CP) score of 7 (CPB7). Unexpectedly, all 9 patients with a CP score of 9 (CPB9) were randomly assigned to the namodenoson group (*p* = 0.0027, chi-squared test) (Table 1).

The median follow-up was 4.1 months. At the time of data analysis, 70 patients had died, 3 were lost to follow-up, and 5 subjects were still under observation for survival. Two of these 5 patients were switched to open-label namodenoson after the blind was broken (per-protocol amendment) after having been treated with blinded namodenoson for 19.2 and 27.6 months, respectively.

**Figure 2:**
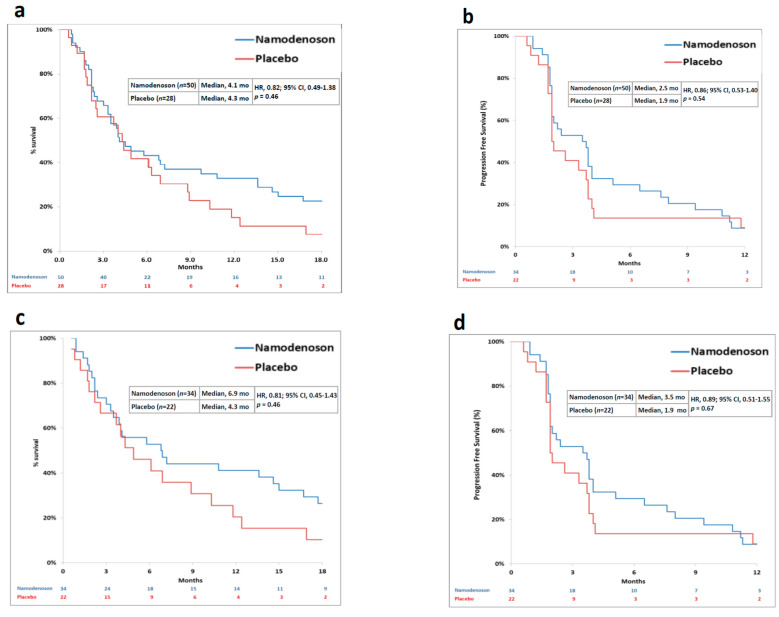
Kaplan–Meier curves by treatment group (namodenoson 25 mg/kg twice a day (BID) and placebo) for all patients and Child–Pugh B7 (CPB7) patients. (**a**) overall survival (OS) in all patients; (**b**) progression-free survival (PFS) in all patients; (**c**) OS in CPB7 patients; (**d**) PFS in CPB7 patients.

The primary endpoint was not met. Median OS was 4.1 months in the namodenoson group and 4.3 months in the placebo group (HR, 0.82; 95% CI: 0.49–1.38, *p* = 0.46) (Figure 2a). Progression-free survival (PFS) was 2.5 months in the namodenoson group and 1.9 months in the placebo group (HR, 0.86; 95% CI: 0.53–1.40, *p* = 0.54) (Figure 2b). The pre-planned exploratory analysis demonstrated that the difference between the arms in 12-month OS rate trended towards significance (32% [16/50] in the namodenoson group versus 14% [4/28] in the placebo group; *p* = 0.058).

Pre-planned analysis evaluated subgroups of patients by their CP score. In CPB7 patients (*n* = 56, 34 namodenoson-treated and 22 placebo-treated), a median OS and PFS were numerically better with namodenoson, (OS: 6.9 versus 4.3 months; HR, 0.81; 95% CI: 0.45–1.43, *p* = 0.46. PFS: 3.5 versus 1.9 months; HR, 0.89; 95% CI: 0.51–1.55, *p* = 0.67) (Figure 2c,d). The 12-month OS rate was statistically significantly better in the namodenoson group (15 out of 34 patients [44%] versus 4 out of 22 patients [18%]; *p* = 0.028). Notably, the other subgroups were small. In patients with CP score of 8 (CPB8) (*n* = 13, 7 namodenoson-treated and 6 placebo-treated), OS and PFS were similar between the treatment arms and shorter than observed in the CPB7 subgroup (OS: 3.3 versus 3.4 months, respectively; HR, 0.88; 95% CI: 0.28–2.77, *p* = 0.83. PFS: 2.1 versus 1.9 months, respectively; HR, 0.71; 95% CI: 0.23–2.17, *p* = 0.53). The median OS and PFS values for the 9 CPB9 patients (all namodenoson-treated) were 3.5 and 2.2 months, respectively, similar to CPB8.

**Figure 3:**
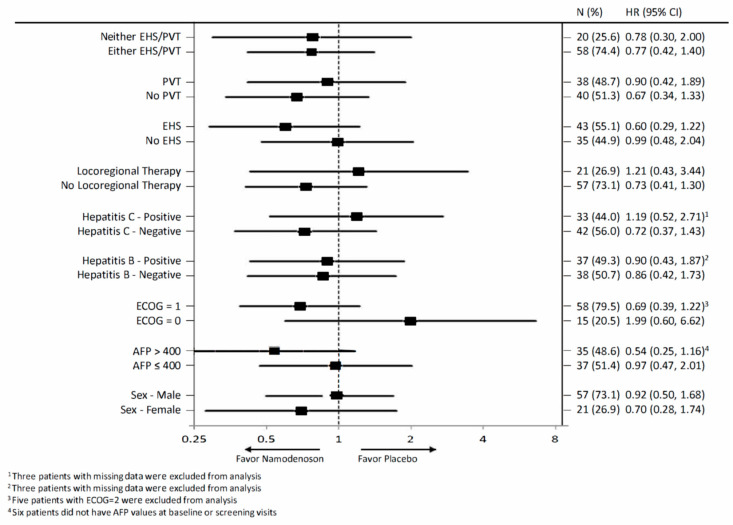
Forest plot summarizing exploratory overall survival (OS) subgroup analysis comparing patients assigned to receive namodenoson 25 mg/kg twice a day (BID) versus placebo. AFP, Alpha-fetoprotein; CI, confidence interval; ECOG PS, Eastern Cooperative Oncology Group performance status; EHS, extrahepatic spread; H, hazard ratio; PVT, portal vein thrombosis.

The exploratory analysis compared OS by treatment arm for various subgroups in the intent-to-treat (ITT) population (Figure 3). The subgroups involved stratification by sex, alpha-fetoprotein (AFP; ≤400, >400), Eastern Cooperative Oncology Group (ECOG) performance status (PS), hepatitis B and C status, locoregional therapy, extrahepatic spread status, and portal vein thrombosis status. None of the subgroups demonstrated a statistically significant difference between the namodenoson and placebo arms.

Response assessment was performed on 55 patients (34 namodenoson-treated, 21 placebo-treated) for whom ≥1 assessment post-baseline was available; assessment could not be performed for the remaining patients due to disease progression or loss to follow-up before an on-treatment evaluation could be performed. Table 2 shows the detailed response information. No patient achieved a complete response (CR). Of the 34 patients assessed in the namodenoson group, 3 (8.8%) achieved partial response (PR) and 17 (50.0%) achieved stable disease (SD) as best response (i.e., disease control rate [DCR] was 58.8%), whereas in the placebo group, of the 21 patients assessed, none achieved PR and 10 (47.6%) achieved SD as the best response (i.e., DCR was 47.6%). In the 3 patients who experienced PR, the duration of response was 2, 6, and 26 months. In an ITT analysis, PR, SD, and DCR rates in the namodenoson group were 6.0%, 34.0%, and 40.0%, respectively, whereas, in the placebo group, the respective rates were 0%, 35.7%, and 35.7%. At the end of both 4 and 6 cycles of treatment, the DCR for namodenoson treatment was statistically superior to that of placebo (ITT analysis; 4 cycles: 18.0% versus 7.1%, *p* = 0.013. 6 cycles: 14.0% versus 7.1%, *p* = 0.038).

Namodenoson was well-tolerated, with a safety profile that was generally comparable to that of placebo (Table 3). No deaths, withdrawals, or dose reductions were attributed to namodenoson. No hepatotoxicity or aberrations of liver function tests were associated with namodenoson administration. Mean serum albumin levels and albumin-bilirubin (ALBI) scores did not change significantly throughout the study period in both arms. Treatment-related adverse events (AEs) are presented in Table 3. There was only one grade 3 treatment-related AE, hyponatremia.

Mean (±SE) A3AR expression level at baseline among study participants was 1.98 ± 0.36 (*n* = 53) versus 1 unit in healthy subjects (*n* = 50). Baseline levels were similar between the namodenoson and placebo arms. In patients who were treated with namodenoson through 12 cycles (*n* = 7), mean (±SE) A3AR at baseline was 2.36 ± 1.53, and at Cycle 12, it was 2.46 ±0.98. There was no correlation between A3AR expression at baseline and tumor response to namodenoson.

## 3. Discussion

Despite recent advances in treatments for advanced HCC with the approval of new drugs for the first-line setting and the second-line setting, patients with CPB cirrhosis still represent unmet clinical need, as current therapies were mostly investigated in CPA patients [12,13,14,15,16,17].

This phase II, double-blind, randomized, placebo-controlled study investigating namodenoson as second-line therapy (after failing sorafenib in the first line) is unique as it enrolled only HCC with underlying CPB cirrhosis. The study enrolled CPB7 (*n* = 56; 34 namodenoson-treated and 21 placebo-treated), CPB8 (*n* = 14, 7 namodenoson-treated and 7 placebo-treated), and CPB9 (*n* = 9, all namodenoson-treated) patients. The study did not meet its primary endpoint, an improvement in OS. The median OS for the 50 subjects treated with namodenoson was 4.1 months versus 4.3 months for the 28 subjects on placebo. Pre-planned subgroup analysis of CPB7 patients showed a median OS of 6.9 versus 4.3 months (HR, 0.81; 95% CI: 0.45–1.43) and a PFS of 3.5 versus 1.9 months (HR, 0.89; 95% CI: 0.51–1.55). Notably, the results may have been distorted due to an imbalance between the study arms with more advanced disease present in the namodenoson arm. The namodenoson arm included a higher proportion of patients who were Barcelona Clinic Liver Cancer (BCLC) stage C compared with the placebo arm (80% versus 64%). Furthermore, all 9 patients with CPB9 cirrhosis, the most severe grade allowed into the trial, were randomly assigned to the namodenoson arm and comprised 18% of that arm. CBP9 patients treated with namodenoson achieved an OS of 3.5 months (versus 6.9 for CBP7, for example), which may have biased the overall results. Two additional findings suggest an efficacy signal of namodenoson over placebo: (a) the increase in 12-month survival in the namodenoson-treated group versus the placebo group; and (b) the 8.8% PR rate in the namodenoson group versus 0% in the placebo group. To the best of our knowledge, very few CPB patients who failed sorafenib have been included in prior prospective clinical trials. The only study which did include 78 CPB patients was phase 3 REACH I study, which evaluated the safety and efficacy of ramucirumab as a second-line treatment. Although the safety profile of ramucirumab in the whole study population was defined as manageable, patients with CPB experienced a higher incidence of grade 3 or higher treatment-emergent AEs [18]. No efficacy response has been recorded with ramucirumab in the CPB patient population [18]. Other therapeutic approaches did demonstrate some effectiveness in CPB patients; however, the evidence is limited to retrospective studies. For example, a real-world cohort study and a case series investigating nivolumab in HCC CBP patients (including 71 and 18 such patients, respectively, mostly in the second-line setting) demonstrated CR rates of 0% and 6%, respectively, and PR rates of 3% and 11%, respectively [19,20]. Metronomic capecitabine (MC) treatment (i.e., chronic administration of capecitabine at low doses without prolonged drug-free breaks) also showed promising results in two retrospective studies of which only one included only HCC CBP patients [21,22]. This retrospective analysis compared MC (*n* = 35) to best supportive care (BSC; *n* = 70) in treatment-naïve CPB patients and demonstrated significantly improved OS with MC vs. BSC (7.5 vs. 5.1 months; *p* = 0.013) [21]. The other MC study involving 26 HCC patients after sorafenib failure demonstrated potential long-term anticancer activity with MC (median time to progression, 4 months); however, only 5 patients (19%) in the case series had CPB cirrhosis [22].

Namodenoson was well-tolerated, with a very favorable safety profile that was generally comparable to that of the placebo group. These results are consistent with the known characteristic of namodenoson, which induces a differential effect on pathological and normal body cells, as manifested by apoptosis of cancer cells and protective effects towards liver cells [8,10]. The findings are also consistent with prior clinical studies in HCC as well as other indications such as nonalcoholic fatty liver disease (NAFLD) and nonalcoholic steatohepatitis (NASH) [11,23]. Thus, the accumulative evidence suggests that namodenoson is a potentially safer alternative to other approved agents in patients with cirrhosis and/or hepatic impairment.

Overexpression of A3AR mRNA has been reported in liver cancer cells as well as in other tumor types (breast, melanoma, prostate, and colon) compared to normal adjacent tissue [6,7]. The high expression also has been observed in the PBMCs of the patients, and it has been demonstrated that it reflects the expression in the tumor cells [6]. It has been further suggested that A3AR expression in PBMCs can serve as a biomarker to predict response to namodenoson; however, since receptor upregulation has been detected in most cancer patients [9], it has been concluded that this approach should not be implemented in cancer treatment. In addition, A3AR belongs to a family of Gi protein associated receptors that respond to agonist treatment by receptor desensitization/re-sensitization [24]. Therefore, the current study also examined A3AR expression levels before and after treatment with namodenoson to evaluate whether the level of A3AR (the namodenoson target) remains stable with chronic treatment. Our findings (albeit with a relatively small sample size) that the receptor overexpression was stable throughout 12 cycles of namodenoson treatment suggest that A3AR is not desensitized upon chronic treatment with namodenoson.

Our study is limited by its sample size and by the imbalance between the study arms with respect to CP score distribution (all 9 patients with CPB9 cirrhosis were in the namodenoson arm).

## 4. Materials and Methods

### 4.1. Study Participants

The study population consisted of patients aged ≥18 years with advanced stage/treatment-refractory HCC and CPB cirrhosis who did not tolerate sorafenib or whose disease had previously progressed on sorafenib treatment. The diagnosis of HCC in subjects without underlying cirrhosis at the time of diagnosis required cytology and/or histology; for subjects with underlying cirrhosis at the time of diagnosis, HCC diagnosis was established according to the American Association for the Study of Liver Diseases practice guidelines algorithm [25]. For subjects who had tolerated sorafenib, ≥3 weeks of prior treatment was required, terminating at ≥2 weeks before study entry. Inclusion criteria included: ECOG PS ≤ 2; having CPB cirrhosis (i.e., CP score 7–9); aspartate aminotransferase (AST) and alanine aminotransferase (ALT) levels ≤ 5 times the upper limit of normal (ULN); total bilirubin, ≤3.0 mg/dL; serum albumin, ≥2.8 g/dL; prothrombin time (PT), <6 s longer than control; serum creatinine, ≤2.0 mg/dL; absolute neutrophil count, ≥1500 × 10^9^/L; and platelet count, ≥75,000 × 10^9^/L. Exclusion criteria included: the presence of hepatic encephalopathy; and gastrointestinal hemorrhage requiring transfusion occurring within the past 4 weeks.

### 4.2. Study Design and Treatment

This was a multicenter, randomized, double-blind, placebo-controlled clinical trial (ClinicalTrials.gov identifier NCT02128958). The trial was conducted at 15 sites in Israel, Europe and the United States.

Subjects were enrolled by the participating centers and were randomly assigned 2:1, using a central randomization schedule generated by an independent biostatistician with no stratification prior to randomization. Patients were randomized to either namodenoson 25 mg or matching placebo, administered every 12 h until discontinuation due to intolerance, withdrawal of consent, or death. Though treatment was administered in a continuous manner, the treatment period was divided into 4-week cycles for the purpose of data recording. No crossover was initially allowed; however, by protocol amendment, patients continuing on blinded treatment were offered open-label namodenoson (25 mg BID) upon unblinding of the treatment assignments. All study site personnel were blinded to patients’ treatment throughout the study period.

The study was approved by all relevant national regulatory authorities and local Ethics Committees/Institutional Review Boards. The study was conducted in accordance with the Declaration of Helsinki, and written informed consent was obtained from all the patients.

### 4.3. Assessments

The primary endpoint of the study was OS. Secondary endpoints were PFS, overall response rate (ORR), disease control rate (DCR, the sum of ORR and SD rates), and safety. As in advanced HCC, PFS was found to be moderately correlated with OS; PFS is a reasonable secondary endpoint in this disease [26]. Disease response assessment was evaluated locally by 2 independent blinded radiologists using Response Evaluation Criteria in Solid Tumors (RECIST) version 1.1 [27]. Tumor status was assessed at baseline and every 8 weeks thereafter by computed tomography scan or magnetic resonance imaging. Safety was monitored through assessments of AEs using the US National Cancer Institute’s Common Terminology Criteria for Adverse Events (CTCAE) v4.03. Changes from baseline in vital signs, clinical laboratory parameters, electrocardiograms, physical examinations, and ECOG PS were also assessed. AFP levels were assessed at baseline and every 4 weeks thereafter, as were laboratory parameters associated with hepatic dysfunction and cirrhosis, such as serum ALT, AST, bilirubin, and albumin levels, PT, and international normalized ratio. ALBI scores were calculated from the albumin and bilirubin levels, as previously described [28].

### 4.4. Biomarker Studies

Another secondary objective was to evaluate the relationship between white blood cell (WBC) A3AR expression (which has been suggested to mirror the expression in HCC tumor cells [6,7,29,30]) as assessed at baseline and every cycle thereafter at selected study centers, (*n* = 53 patients) and clinical response. A3AR mRNA expression in WBC was determined from blood collected to a PAXgene RNA tube (Qiagen, Venlo, The Netherlands), using the QuantiGene Plex 2.0^®^ assay (Thermo Fisher, Waltham, MA, USA). β-actin was used as a reference control, and the oligonucleotide probe sets were designed by Thermo Fisher. Luminescence from each specific probe set was captured by GloMax Multi (Promega, Madison, WI, USA). A3AR was expressed in units, where 1 unit was defined as the mean of A3AR expression in healthy subjects (*n* = 50). Healthy subjects were 20–70 years of age with no known illness and no prior treatment.

### 4.5. Statistical Analysis

Power calculation determined that 75 deaths, assuming a hazard ratio of 0.5, would provide 80% power for the log-rank test at a significance level of 0.05. Primary efficacy analyses were performed on the ITT population. Descriptive statistics were used to summarize patient/tumor characteristics and safety. Kaplan–Meier curves were used to estimate OS/PFS, and between comparisons were performed using log-rank tests. The Cox proportional hazards regression model was used to assess the impact of covariates. ORR/DCR were determined using the normal approximation to the binomial distribution by treatment. The statistical analysis plan was amended prior to unblinding to include subgroup analysis by CP score. All statistical tests were 2-sided, and *p* < 0.05 was considered statistically significant. Statistical analyses were conducted using SAS 9.4 (SAS Institute Inc., Cary, NC, USA).

## 5. Conclusions

Although the study did not meet its primary endpoint, our findings demonstrate a favorable safety profile of namodenoson and suggest a positive efficacy signal in HCC CPB7 patients. As the sample size in our study was small, the results should be confirmed in a larger study.

## Figures and Tables

**Table 1:** Baseline patient and tumor characteristics.

Characteristic	Namodenoson *n* = 50	Placebo *n* = 28
Age, median (range), years	62 (24–81)	66 (41–83)
Gender, *n* (%)		
Male	38 (76.0%)	19 (67.6%)
Female	12 (24.0%)	9 (32.1%)
Ethnicity, *n* (%)		
White/Caucasian	48 (96.0%)	27 (96.4%)
Black/African	1 (2.0%)	0 (0.0%)
Asian	0 (0.0%)	1 (3.6%)
Other	1 (2.0%)	0 (0.0%)
Child–Pugh score, *n* (%)		
7	34 (68.0%)	22 (78.6%)
8	7 (14.0%)	6 (21.4%)
9	9 (18.0%)	0 (0.0%)
BCLC stage, *n* (%)		
B	10 (20.0%)	10 (35.7%)
C	40 (80.0%)	18 (64.3%)
Hepatitis status, *n* (%)		
None	13 (26.0%)	11 (39.2%)
Hepatitis B	19 (38.0%)	10 (35.7%)
Hepatitis C	25 (50.0%)	9 (32.1%)
Hepatitis B and C	7 (14.0%)	2 (7.1%)
ECOG PS, *n* (%)		
0	10 (20.0%)	5 (17.9%)
1	37 (74.0%)	21 (75.0%)
2	3 (6.0%)	2 (7.1%)
AFP, *n* (%)		
≤400 ng/mL	22 (44.0%)	15 (53.6%)
>400 ng/mL	23 (46.0%)	12 (42.9%)
N/A	5 (10.0%)	1 (3.6%)
PVT, *n* (%)		
Yes	26 (52.0%)	12 (42.9%)
No	24 (48.0%)	16 (57.1%)
EHS, *n* (%)		
Yes	29 (58.0%)	14 (50.0%)
No	21 (42.0%)	14 (50.0%)
Prior therapy (chemoembolization), *n* (%)		
Yes	15 (30.0%)	6 (21.4%)
No	35 (70.0%)	22 (78.6%)

Abbreviations: AFP, alpha-fetoprotein; BCLC, Barcelona Clinic Liver Cancer; ECOG, Eastern Cooperative Oncology Group; EHS, extrahepatic spread; N/A, not available; PS, performance status; PVT, portal vein thrombosis.

**Table 2:** Best observed responses (RECIST 1.1) by treatment arm.

Response, *n* (%)	Namodenoson *n* = 34 ^1^	Placebo *n* = 21 ^1^
CR	0 (0.0%)	0 (0.0%)
PR	3 (8.8%)	0 (0.0%)
SD	17 (50.0%)	10 (47.6%)
PD	14 (41.2%)	11 (52.4%)

Abbreviations: CR, complete response; ITT, intent-to-treat; PD, progressive disease; PR, partial response; RECIST, response evaluation criteria in solid tumors; SD, stable disease. ^1^ Patients with no assessment post-baseline due to disease progression or loss to follow-up were excluded from the analysis. In an ITT analysis, the PR/SD/PD rates in the namodenoson arm were 6.0%/34.0%/28.0%; and for the placebo arm, these rates were 0%/35.7%/39.3%.

**Table 3:** Treatment-related adverse events.

Adverse Event	Grade 1–2		Grade 3	
	Namodenoson *n* = 50	Placebo *n* = 28	Namodenoson *n* = 50	Placebo *n* = 28
Any	10 (20.0%)	14 (50.0%)	1 (2.0%)	1 (3.6%)
Abdominal pain	1 (2.0%)			
Anemia	1 (2.0%)	1 (3.6%)		1 (3.6%)
Asthenia/fatigue		3 (10.7%)		1 (3.6%)
Bronchitis		1 (3.6%)		
Chest pain	1 (2.0%)	1 (3.6%)		
Diarrhea	1 (2.0%)	1 (3.6%)		
Dyspepsia	1 (2.0%)			
Hypoesthesia	1 (2.0%)			
Hyponatremia			1 (2.0%)	
Hypotension	1 (2.0%)			
Nausea	2 (4.0%)	1 (3.6%)		
Peripheral edema		1 (3.6%)		
Paresthesia	1 (2.0%)			
Pyrexia		1 (3.6%)		
Sinus tachycardia		1 (3.6%)		
Vomiting	1 (2.0%)	1 (3.6%)		
Weight decreased/abnormal weight loss	2 (4.0%)			
Weight increased	2 (4.0%)	2 (7.1%)		
ALT Increased	1 (2.0%)			
Creatinine increased		1 (3.6%)		
INR abnormal		1 (3.6%)		
Leukopenia	1 (2.0%)			
Neutropenia	1 (2.0%)			
Thrombocytopenia	1 (2.0%)			
Increased TSH	1 (2.0%)	2 (7.1%)		
Decreased T_3_		1 (3.6%)		

Abbreviations: ALT, alanine aminotransferase; INR, international normalized ratio; TSH, thyroid-stimulating hormone.

## Data Availability

The data presented in this study are available on request from the corresponding author. The data are not publicly available due to privacy concerns.

## References

[1] Fitzmaurice C., Akinyemiju T.F., Al Lami F.H., Alam T., Alizadeh-Navaei R., Allen C., Alsharif U., Alvis-Guzman N., Amini E., Global Burden of Disease Cancer Collaboration (2018). Global, regional, and national cancer incidence, mortality, years of life lost, years lived with disability, and disability-adjusted life-years for 29 cancer groups, 1990 to 2016: A systematic analysis for the global burden of disease study. JAMA Oncol..

[2] Granito A., Bolondi L. (2017). Non-transplant therapies for patients with hepatocellular carcinoma and child-pugh-turcotte class b cirrhosis. Lancet Oncol..

[3] FDA Website Sorafenib Package Insert. https://www.accessdata.fda.gov/drugsatfda_docs/label/2018/021923s020lbl.pdf..

[4] Llovet J.M., Di Bisceglie A.M., Bruix J., Kramer B.S., Lencioni R., Zhu A.X., Sherman M., Schwartz M., Lotze M., Talwalkar J. (2008). Design and endpoints of clinical trials in hepatocellular carcinoma. J. Natl. Cancer Inst..

[5] NIH U.S. National Library of Medicine, Clinical Trials Data Base. ClinicalTrials.gov.

[6] Bar-Yehuda S., Stemmer S.M., Madi L., Castel D., Ochaion A., Cohen S., Barer F., Zabutti A., Perez-Liz G., Del Valle L. (2008). The A3 adenosine receptor agonist CF102 induces apoptosis of hepatocellular carcinoma via de-regulation of the Wnt and NF-kappaB signal transduction pathways. Int. J. Oncol..

[7] Madi L., Ochaion A., Rath-Wolfson L., Bar-Yehuda S., Erlanger A., Ohana G., Harish A., Merimski O., Barer F., Fishman P. (2004). The A3 adenosine receptor is highly expressed in tumor versus normal cells: Potential target for tumor growth inhibition. Clin. Cancer Res..

[8] Cohen S., Stemmer S.M., Zozulya G., Ochaion A., Patoka R., Barer F., Bar-Yehuda S., Rath-Wolfson L., Jacobson K.A., Fishman P. (2011). CF102 an A3 adenosine receptor agonist mediates anti-tumor and anti-inflammatory effects in the liver. J. Cell Physiol..

[9] Fishman P., Bar-Yehuda S., Synowitz M., Powell J., Klotz K., Gessi S., Borea P., Wilson C.N., Mustafa S.J. (2009). Adenosine receptors and cancer. Health and Disease, Handbook of Experimental Pharmacology.

[10] Fishman P., Bar-Yehuda S., Barer F., Madi L., Multani A.S., Pathak S. (2001). The A3 adenosine receptor as a new target for cancer therapy and chemoprotection. Exp. Cell Res..

[11] Stemmer S.M., Benjaminov O., Medalia G., Ciuraru N.B., Silverman M.H., Bar-Yehuda S., Fishman S., Harpaz Z., Farbstein M., Cohen S. (2013). CF102 for the treatment of hepatocellular carcinoma: A phase I/II, open-label, dose-escalation study. Oncologist.

[12] FDA Website Cabozantinib Package Insert. https://www.accessdata.fda.gov/drugsatfda_docs/label/2019/208692s003lbl.pdf.

[13] FDA Website Regorafenib Package Insert. https://www.accessdata.fda.gov/drugsatfda_docs/label/2012/203085lbl.pdf.

[14] El-Khoueiry A.B., Sangro B., Yau T., Crocenzi T.S., Kudo M., Hsu C., Kim T.Y., Choo S.P., Trojan J., Welling T.H.R. (2017). Nivolumab in patients with advanced hepatocellular carcinoma (CheckMate 040): An open-label, non-comparative, phase 1/2 dose escalation and expansion trial. Lancet.

[15] Finn R.S., Ryoo B.Y., Merle P., Kudo M., Bouattour M., Lim H.Y., Breder V., Edeline J., Chao Y., Ogasawara S. (2020). Pembrolizumab as second-line therapy in patients with advanced hepatocellular carcinoma in Keynote-240: A randomized, double-blind, phase III trial. J. Clin. Oncol..

[16] FDA Website Ramucirumab Package Insert. https://www.accessdata.fda.gov/drugsatfda_docs/label/2014/125477s002lbl.pdf.

[17] Finn R.S., Qin S., Ikeda M., Galle P.R., Ducreux M., Kim T.Y., Kudo M., Breder V., Merle P., Kaseb A.O. (2020). Atezolizumab plus bevacizumab in unresectable hepatocellular carcinoma. N. Engl. J. Med..

[18] Zhu A.X., Baron A.D., Malfertheiner P., Kudo M., Kawazoe S., Pezet D., Weissinger F., Brandi G., Barone C.A., Okusaka T. (2017). Ramucirumab as second-line treatment in patients with advanced hepatocellular carcinoma: Analysis of REACH trial results by Child-Pugh score. JAMA Oncol..

[19] Choi W.M., Lee D., Shim J.H., Kim K.M., Lim Y.S., Lee H.C., Yoo C., Park S.R., Ryu M.H., Ryoo B.Y. (2020). Effectiveness and safety of nivolumab in child-pugh b patients with hepatocellular carcinoma: A real-world cohort study. Cancers.

[20] Kambhampati S., Bauer K.E., Bracci P.M., Keenan B.P., Behr S.C., Gordan J.D., Kelley R.K. (2019). Nivolumab in patients with advanced hepatocellular carcinoma and child-pugh class b cirrhosis: Safety and clinical outcomes in a retrospective case series. Cancer.

[21] De Lorenzo S., Tovoli F., Barbera M.A., Garuti F., Palloni A., Frega G., Garajova I., Rizzo A., Trevisani F., Brandi G. (2018). Metronomic capecitabine vs. best supportive care in child-pugh b hepatocellular carcinoma: A proof of concept. Sci. Rep..

[22] Granito A., Marinelli S., Terzi E., Piscaglia F., Renzulli M., Venerandi L., Benevento F., Bolondi L. (2015). Metronomic capecitabine as second-line treatment in hepatocellular carcinoma after sorafenib failure. Dig. Liver Dis..

[23] Safadi R., Braun M., Milgrom Y., Masarowa M., Hakimian D., Hazou W., Issacchar A., Harpaz Z., Farbstein M., Itzhak I. A phase 2, randomized, double-blind, placebo-controlled dose-finding study of the efficacy and safety of namodenoson (CF102), an A3 Adenosine Receptor (A3AR) agonist, in treating non-alcoholic fatty liver disease (NAFLD) and non-alcoholic steatohepatitis (NASH). Proceedings of the The Liver Meeting Digital Experience™, Digital Conference.

[24] Bunemann M., Lee K.B., Pals-Rylaarsdam R., Roseberry A.G., Hosey M.M. (1999). Desensitization of G-protein-coupled receptors in the cardiovascular system. Annu. Rev. Physiol..

[25] Marrero J.A., Kulik L.M., Sirlin C.B., Zhu A.X., Finn R.S., Abecassis M.M., Roberts L.R., Heimbach J.K. (2018). Diagnosis, staging, and management of hepatocellular carcinoma: 2018 practice guidance by the American Association for the Study of Liver Diseases. Hepatology.

[26] Llovet J.M., Montal R., Villanueva A. (2019). Randomized trials and endpoints in advanced HCC: Role of PFS as a surrogate of survival. J. Hepatol..

[27] Eisenhauer E.A., Therasse P., Bogaerts J., Schwartz L.H., Sargent D., Ford R., Dancey J., Arbuck S., Gwyther S., Mooney M. (2009). New response evaluation criteria in solid tumours: Revised RECIST guideline (version 1.1). Eur. J. Cancer.

[28] Johnson P.J., Berhane S., Kagebayashi C., Satomura S., Teng M., Reeves H.L., O’Beirne J., Fox R., Skowronska A., Palmer D. (2015). Assessment of liver function in patients with hepatocellular carcinoma: A new evidence-based approach-the ALBI grade. J. Clin. Oncol..

[29] Gessi S., Cattabriga E., Avitabile A., Gafa R., Lanza G., Cavazzini L., Bianchi N., Gambari R., Feo C., Liboni A. (2004). Elevated expression of A3 adenosine receptors in human colorectal cancer is reflected in peripheral blood cells. Clin. Cancer Res..

[30] Fishman P., Bar-Yehuda S., Ardon E., Rath-Wolfson L., Barrer F., Ochaion A., Madi L. (2003). Targeting the A3 adenosine receptor for cancer therapy: Inhibition of prostate carcinoma cell growth by A3AR agonist. Anticancer Res..

